# Associations between vertebral fractures, increased thoracic kyphosis, a flexed posture and falls in older adults: a prospective cohort study

**DOI:** 10.1186/s12877-015-0018-z

**Published:** 2015-03-28

**Authors:** Hanna C van der Jagt-Willems, Maartje H de Groot, Jos PCM van Campen, Claudine JC Lamoth, Willem F Lems

**Affiliations:** Department of Geriatric Medicine, Slotervaart Hospital, Amsterdam, The Netherlands; Department of Internal Medicine and Geriatrics, Academic Medical Center, Amsterdam, The Netherlands; School of Health, Saxion University of Applied Sciences, Enschede, The Netherlands; University of Groningen, University Medical Center Groningen, Center for Human Movement Sciences, Groningen, The Netherlands; Department of Rheumatology, VU Medical Center, Amsterdam, The Netherlands

**Keywords:** Falls, Thoracic kyphosis, Vertebral fractures, Flexed posture, Older adults

## Abstract

**Background:**

Vertebral fractures, an increased thoracic kyphosis and a flexed posture are associated with falls. However, this was not confirmed in prospective studies. We performed a prospective cohort study to investigate the association between vertebral fractures, increased thoracic kyphosis and/or flexed posture with future fall incidents in older adults within the next year.

**Methods:**

Patients were recruited at a geriatric outpatient clinic. Vertebral fractures were evaluated on lateral radiographs of the spine with the semi-quantitative method of Genant; the degree of thoracic kyphosis was assessed with the Cobb angle. The occiput-to-wall distance was used to determine a flexed posture. Self-reported falls were prospectively registered by monthly phone contact for the duration of 12 months.

**Results:**

Fifty-one older adults were included; mean age was 79 years (SD = 4.8). An increased thoracic kyphosis was independently associated with future falls (OR 2.13; 95% CI 1.10-4.51). Prevalent vertebral fractures had a trend towards significancy (OR 3.67; 95% CI 0.85-15.9). A flexed posture was not significantly associated with future falls.

**Conclusion:**

Older adults with an increased thoracic kyphosis are more likely to fall within the next year. We suggest clinical attention for underlying causes. Because patients with increased thoracic curvature of the spine might have underlying osteoporotic vertebral fractures, clinicians should be aware of the risk of a new fracture.

## Background

Among older adults the prevalence of falls is high: at least 30-40% of patients aged over 65 experience one or more fall accidents annually [1]. Falls in the older population are generally caused by a combination of risk factors, such as balance and gait disorders, poor vision, polypharmacy and environmental factors, and could lead to serious injuries such as fractures [2]. In addition, diminished bone quality due to osteoporosis increases the risk of fall-related fractures, especially in women [3,4]. However, typical osteoporotic fractures of the vertebrae are commonly not the result of a fall incident, but occur usually during normal activities of daily living, such as climbing stairs, lifting groceries, or bending forward [5]. The prevalence of vertebral fractures increases with age, and is up to 50% among geriatric outpatients [6]. Vertebral fractures could cause pain and may lead to postural changes, restrictive respiratory disease, poor physical condition, and loss of quality of life [7], and are independently associated with increased mortality [8,9]. Furthermore, it was recently found that a prevalent vertebral fracture on a chest computed tomography (CT) was associated with a threefold increased risk of a future hip fracture [10].

Over time, thoracic vertebral fractures could increase the kyphotic curvature of the thoracic spine [11], and may therefore cause a flexed posture [12]. A flexed posture is characterized by an increased thoracic kyphosis, protrusion of the head, and in more severe cases also hip and knee flexion. A flexed posture is the more extreme expression of an increased thoracic kyphosis, when the compensatory mechanisms to correct the kyphosis fail [12].

Previous studies showed that the presence of both vertebral fractures and an increased thoracic kyphosis are related with increased fall risk [13-15]. Recently, we showed that vertebral fractures, increased thoracic kyphosis and a flexed posture are associated with an impaired postural control [16,17]. Patients that have one or more of these entities present might fall more often, since impairments in balance and gait are the primary cause of falls [18]. Until now, this association has not been prospectively investigated. Therefore, we performed a prospective cohort study to investigate the association between prevalent vertebral fractures, increased thoracic kyphosis and a flexed posture with future fall incidents in older adults.

## Methods

### Patient characteristics

The study population comprised visitors of the geriatric outpatient clinic of the Slotervaart Hospital in Amsterdam between October 2010 and April 2012. They were referred to the clinic for various reasons, including memory complaints, mobility problems, or reducing polypharmacy. Eligible patients should be 70 years or older; should be able to walk safely for 3 minutes without using any assistive device (e.g. walking stick or wheeler); and should be able to understand and speak Dutch or English. Patients were excluded if they had any mobility problems due to (lateral) neurological or orthopedic disorders with function limitations of one or both legs; or if they were unable to understand the instructions of the researcher due to severe cognitive or hearing impairments.

All patients received a comprehensive geriatric assessment [18], being standard procedure at the geriatric outpatient clinic. Depending on the conclusions of the geriatrician, work up treatment was started. If the patient was referred to this clinic for fall-related problems, or reported falls in the last year, the Dutch national guidelines for the prevention of falls were followed [19]. The present study was approved by the Medical Ethical Committee of the Slotervaart Hospital and Reade. All patients (or their legal representatives) gave their informed consent.

### Data collection

Gender, age, Body Mass Index (BMI), number of prescriptions, hip replacement in history, and self-reported falls in the last year were recorded. The presence of comorbid diseases was scored using the Charlson Comorbidity Index (CCI) [20]. Cognitive functioning was examined by the Mini Mental State Examination (MMSE) [21].

The prevalence of vertebral fractures was assessed on standing upright lateral X-rays of the thoracic and lumbar vertebral spine. Vertebral fractures were scored by the semi-quantitative technique of Genant [22,23]. All radiographs were scored by two observers (MG and HJ). Their conclusions were compared, and if they differed, final consensus was reached by discussion.

The kyphosis of the thoracic vertebral column was determined by the Cobb angle, the angle between the superior endplate of the second, and the inferior endplate of the twelfth thoracic vertebra, as measured on the same lateral X-ray of the thoracic spine as was used for the judgment of vertebral fractures. In the present study, hyperkyphosis was defined as a Cobb angle of ≥50° [24], a Cobb angle <50° was considered normal. Two observers (MG and HJ) measured the Cobb angle twice, and the mean value of the four measurements was used.

**Figure 1:**
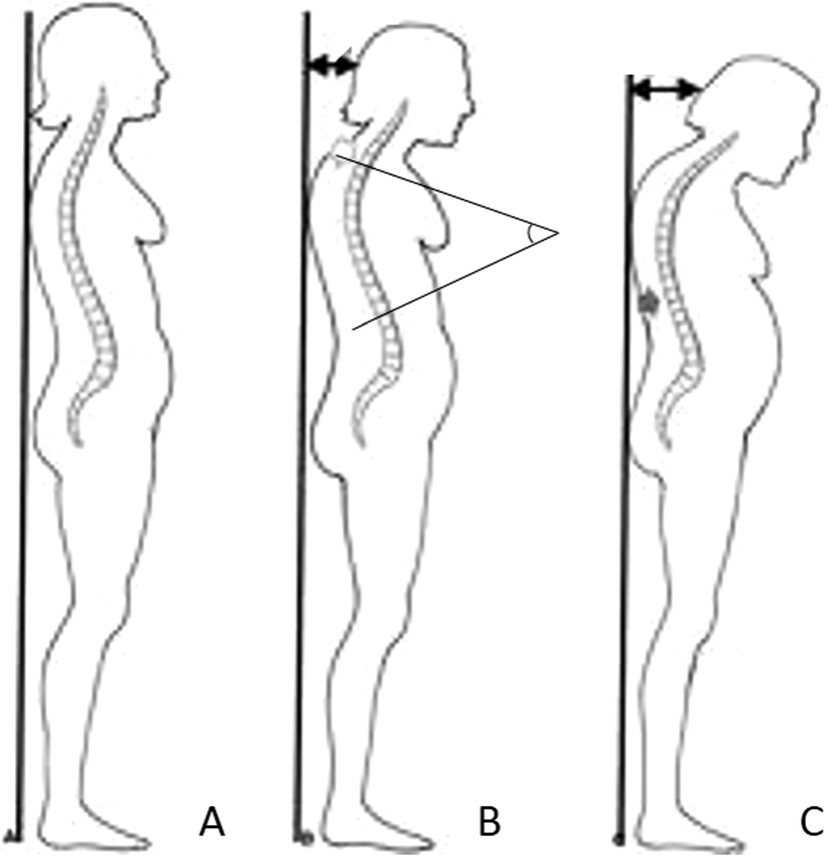
**Various postures among geriatric patients. (A)** Normal posture; **(B)** Hyperkyphosis, defined as a Cobb angle ≥50 ° between T2 and T12 as measured on the X-ray of the vertebral column; **(C)** Flexed posture, defined as an occiput-to-wall distance >5.0 cm.

The severity of flexed posture was evaluated by measuring the occiput-to-wall distance (OWD), see Figure 1. While subjects stood with their head in a natural position, their heels and back touching the wall and their knees as extended as possible, the distance between their occiput and the wall was measured [12]. A flexed posture was defined as an OWD >5.0 cm.

Fall incidents were prospectively registered for six months using a monthly calendar. A fall was defined as “an unexpected event where a person comes to rest on the ground from an upper level or the same level” [25]. During follow-up, patients (or their caregivers) were contacted by phone every month to report any fall incidents and/or injuries. When patients had a MMSE-score <24 points, caregivers who lived with the patient were asked to fill in the falls-and-fracture calendar. Since very few fall incidents were reported in the six-month follow-up period, we extended the falls-and fracture calendar with another six months. Main outcome of the study was the first fall during follow-up.

### Statistical analysis

The associations among vertebral fractures, hyperkyphosis (Cobb angle ≥50°) and flexed posture (OWD >5.0 cm) were estimated using Chi-square tests. Then, to test which variables were associated with prospective falls, first univariate binary logistic analyses were performed for all patient characteristics. Secondly, a multivariate binary logistic regression analysis was computed (method: backward conditional), including all characteristics with P values < .20 in univariate analyses. Odds Ratio’s (OR) with 95% Confidence Intervals (CI) and P values were calculated. In order to better compare the ORs of the variables, we standardized all continuous variables to unit variance, i.e., means were set to zero, and standard deviations to one (*z*-transformation). In addition, to test for multicollineartiy, we calculated the variance inflation factor (VIF) for the multivariate model. Since all VIF-values were around 1, we can assume that multicollinearity was not present in the model. For all statistical analyses, the level of significance was set on P < .05. SPSS Statistics version 21 was used.

## Results

During the inclusion period, 139 possibly eligible patients visited the geriatric, whereof 60 persons gave their consent to participate in the present study. In nine cases, the falls-and-fracture calendar was not completed, due to lost to follow-up within the first month. Finally, 51 patients were included in the present study. The mean age of the included patients was 79 years, and 77% were female (Table 1). Seventeen patients (33%) reported ≥2 falls in the year previous to the baseline measurements.

**Table 1:** **Population characteristics (** ***n*** **= 51)**

**Population characteristics**	
*Patient characteristics*	
Age (years), mean (SD)	79.3 (4.8)
Female, *n* (%)	39 (77%)
BMI (kg/m^2^), mean (SD)	27.4 (4.0)
CCI score, mean (SD)	1.4 (1.3)
Number of prescriptions, mean (SD)	5.8 (3.9)
MMSE score, median (range)	24 (13–30)
Hip replacement in history, *n* (%)	7 (14%)
*Osteoporosis-related factors*	
Presence of vertebral fractures, *n* (%)	20 (39%)
Thoracic kyphosis, Cobb angle (°), mean (SD)	51.2 (14.5)
OWD (cm), median (range)	4.0 (0–16)
*Falls during follow-up*	
no falls, *n* (%)	38 (74%)
1 fall, *n* (%)	5 (10%)
≥2 falls during follow-up (range 2–9), *n* (%)	8 (16%)

**CCI** = Charlson Comorbidity Index; **MMSE** = Mini-Mental State Examination; **OWD** = Occiput-to-Wall Distance.

The mean follow-up for falls registration was 10.6 months. Thirty-eight patients (75%) had follow-up of twelve months with phone contact every month; the other thirteen patients had a mean follow-up of 6.2 months. After the first six months, eight patients refused further follow-up. Other reasons for lost to follow-up were: moving to a nursing home (*n* = 3); and being tired of registering high fall incidence (*n* = 2).

Thirteen patients (25%) had at least one fall during follow-up; of these, eight patients were recurrent fallers (≥2 falls). Four patients had serious injury after the fall and had to visit a doctor, of whom one had a new non-vertebral fracture.

### Relation between vertebral fractures, hyperkyphosis and flexed posture

**Figure 2:**
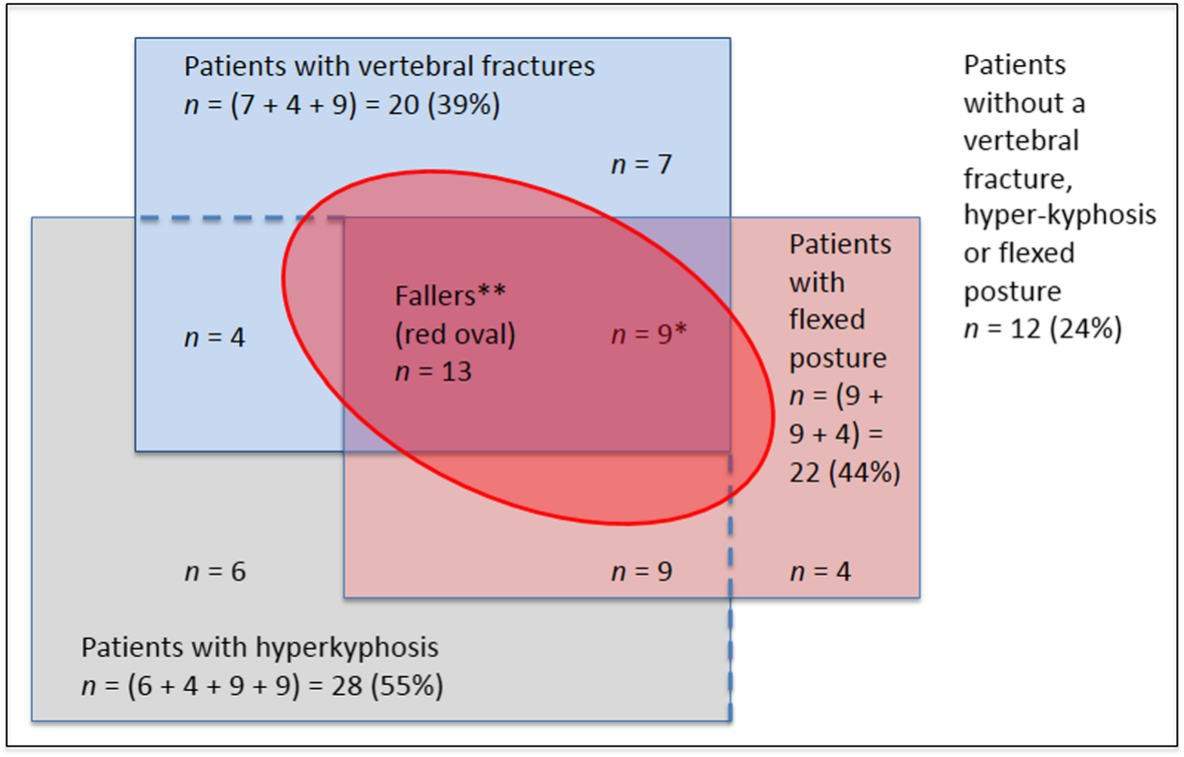
**Illustration of the distribution of patients in the study according to the presence of vertebral fractures, hyperkyphosis and flexed posture in relation to future falls.** The large white rectangle represents all patients in the study (*n* = 51), whereof in the blue rectangle patients with vertebral fractures (*n* = 20; 39%); in the grey rectangle patients with a hyperkyphosis (Cobb angle ≥50 °; *n* = 28; 55%); and in the pink rectangle patients with a flexed posture (OWD >5.0 cm; *n* = 22; 44%). Patients with combinations of these entities are represented by the overlapping areas of the colored rectangles, with *n* noted in each box. Twelve patients (24%) had none of the entities present (white rectangle). * 9 patients had all entities present. ** The red oval represents all fallers (*n* = 13); all fallers had at least one of the three entities present. In nine fallers all entities were present.

Figure 2 shows the distribution of vertebral fractures, hyperkyphosis (Cobb angle ≥50°), and flexed posture (OWD >5.0 cm) in the study population. Twelve patients had (24%) none of the three entities. Nine patients (18%) were diagnosed with all three entities. The remaining thirty patients (59%) had one, or a combination of the entities.

Of the twenty patients with one or more vertebral fractures, thirteen (65%) had also a hyperkyphosis (*χ*^2^ = 1.36; P = .24). Hyperkyphosis was significantly associated with the presence of a flexed posture (*χ*^2^ = 11.32; P < .01). The association between flexed posture and vertebral fractures was not significant (*χ*^2^ = 0.47; P = .83).

### Association of vertebral fractures, thoracic kyphosis and occiput-to-wall distance with future falls

In the univariate analyses (see Table 2), a significant association was found between the Cobb angle and future falls (OR 2.07; 95% CI 1.03-4.16). The presence of one or more vertebral fractures had a trend toward a significant association with future falls (OR 3.47; 95% CI 0.94-12.8). The OWD was not significantly related with prospective fall incidents (OR 1.54; 95% CI 0.82-2.91).

**Table 2:** **Univariate and multivariate associations of the patient characteristics with future falls**

	**Non-fallers**	**Fallers**	**Univariate analyses**		**Multivariate analysis** ^**a**^	
**Patient characteristics**	**(n = 38)**	**(n = 13)**	**OR (95% CI)**	**P value**	**OR (95% CI)**	**P value**
Age (years), mean (SD)	79.7 (4.7)	78.0 (5.0)	0.70 (0.37-1.32)	.27		
Female, n (%)	28 (74%)	11 (85%)	1.96 (0.37-10.4)	.43		
BMI (kg/m^2^), mean (SD)	27.3 (3.9)	27.7 (4.3)	1.12 (0.58-2.16)	.73		
CCI (score), median (SD)	1 (0–4)	1 (0–5)	1.26 (0.68-2.34)	.47		
Number of prescriptions, median (range)	4.5 (0–15)	7 (0–13)	1.51 (0.81-2.80)	.19	1.86 (0.91-3.79)	.09
MMSE (score), median (range)	24 (15–30)	23 (13–28)	0.82 (0.44-1.52)	.53		
Hip replacement in history, n (%)	5 (13%)	2 (15%)	1.20 (0.20-1.09)	.84		
Vertebral fractures, n (%)	12 (32%)	8 (62%)	3.47 (0.94-12.8)	.06	3.67 (0.85-15.9)	.08
Cobb angle (°), mean (SD)	49 (13)	59 (16)	2.07 (1.03-4.16)	.04	2.13 (1.10-4.51)	.04
OWD (cm), mean (SD)	4.2 (4.5)	6.2 (4.1)	1.54 (0.82-2.91)	.18		

All continuous variables were first *z*-transformed.

**BMI** = Body Mass Index; **CCI** = Charlson Comorbidity Index; **CI** = Confidence Interval; **MMSE** = Mini-Mental State Examination; **OR** = Odds Ratio; **OWD** = Occiput-to-Wall Distance.

^a^Adjusted for vertebral fractures, the Cobb angle, the OWD, and the number of prescriptions. After step 1, the OWD was excluded.

In the multivariate analysis (Table 2), including all characteristics with P < .20 in the univariate analysis, only the Cobb angle was independently associated with falls during follow-up (OR 2.13; 95% CI: 1.10-4.51). This indicates that for every standard deviation increase in the Cobb angle, the probability of a future fall doubles. Furthermore, the presence of vertebral fractures, and the number of prescriptions showed a trend towards significance for future falls in our study population.

## Discussion

We showed that in this population an increased thoracic kyphosis, as measured by the Cobb angle, was independently associated with future fall incidents (standardized OR: 2.13; 95% CI: 1.10-4.51). We did not find a significant association between a flexed posture, as indicated by the OWD, and falls. In the multivariate analysis, the presence of one or more vertebral fractures and future falls had a trend towards a significant association (P = .08).

It is remarkable that the presence of an increased thoracic kyphosis had such a clear association with future falls, even in this small study sample. This is in coincidence with previous retrospective studies [13-15]. The Rancho-Bernardo study (*n* = 1883) [14,15], for instance, showed that men with hyperkyphotic posture had an independent age-adjusted association with self-reported falls in the past year. In women, this association appeared to be age-dependent. In addition, a smaller study (*n* = 92) showed that kyphosis of the total spine was independently associated with self-reported falls [13].

An increased thoracic kyphosis can originate from many causes, such as vertebral fractures, degenerative disc diseases, muscle weakness, and genetic disorders such as Scheuermanns disease [12,26]. The prevalence of vertebral fractures in patients with hyperkyphosis (39%) in our study was equal compared to other studies [27-29]. In these studies also no relation was found between vertebral fractures and hyperkyphosis [27-29]. However, the consequences of hyperkyphosis in the long term may be severe, even without vertebral fractures. One study showed that older women with greater degrees of kyphosis are at increased risk of non-spinal fractures, independent of bone mineral density and vertebral fractures [11]. In addition, patients with more severe kyphosis experienced more decline in functioning occurs during a long-term follow-up [30]. Therefore, clinicians should be alert of the presence of vertebral fractures in patients with hyperkyphosis and provide adequate treatment to prevent subsequent fractures. In addition, several studies have shown promising improvements in kyphosis with, amongst others, 6-month spinal bracing intervention [31], 12-week yoga intervention [32], and 12 weeks of multidimensional group exercise [33].

In the present study, fall incidence was lower than expected, namely 26% where at least 30% was expected based on the literature [2]. This low fall rate was remarkable, because our participants were relatively old (mean age: 79 years) and had many fall risk factors such as a history of falls in the past year, substantial co–morbidity. The low fall incidence in our cohort might be the result of a successful visit to our geriatric outpatient clinic, where fall risk was analyzed, and various advices were given to minimize the chance of a fall accident according to the Dutch guidelines [19]. Otherwise, although we gave a calendar and called patients every month, there might be underreporting: it is known that falls, even in healthy older adults, are easily forgotten [34].

Some limitations of this study should be addressed. Because of the small sample size and the low fall incidence during follow-up, we might have overestimated the independent association between the Cobb angle and future falls. Our standardized OR of 2.13 is larger than reported OR’s in studies with more participants [14,15]. Moreover, the association between vertebral fractures and prospective falls was quite strong, but not significant (P = .08). This might be a type II error, namely the failure to reject the false null hypothesis, caused by the low sample size, and therefore the large confidence interval. The relatively high OR of 3.67 shows the importance of vertebral fractures in relation to future falls. Future larger studies between older adults should investigate the association between vertebral fractures and falls, since this was not investigated before.

However, despite these limitations, we can conclude that there is a clear independent association between an increased thoracic curvature of the spine and future falls. An explanation might be that due to the forward curvature of the upper body, the center of mass shifts forward and requires correcting responses of the body. These correcting responses may reduce the patient’s ability to respond on perturbations, which is reflected by an impaired postural control during walking as found in previous studies [16,17].

## Conclusions

Since older adults with a hyperkyphosis may thus have an increased fall risk, as we show in this study, we suggest clinical attention for these patients to search for underlying causes. In almost 40% of the patients with hyperkyphosis in our cohort, one or more vertebral fractures were present. For these patients, we therefore should not forget to prescribe anti-osteoporosis medication. Future research should further evaluate whether hyperkyphosis is an important risk factor for falls and which therapies may prevent, improve or delay its progression.
